# A Potential Compensatory Role of Panx3 in the VNO of a Panx1 Knock Out Mouse Model

**DOI:** 10.3389/fnmol.2018.00135

**Published:** 2018-04-26

**Authors:** Paige Whyte-Fagundes, Stefan Kurtenbach, Christiane Zoidl, Valery I. Shestopalov, Peter L. Carlen, Georg Zoidl

**Affiliations:** ^1^Department of Biology, York University, Toronto, ON, Canada; ^2^Krembil Research Institute, University Health Network, Toronto, ON, Canada; ^3^Department of Ophthalmology, Bascom Palmer Eye Institute, University of Miami Miller School of Medicine, Miami, FL, United States; ^4^Department of Physiology, University of Toronto, Toronto, ON, Canada; ^5^Department of Psychology, York University, Toronto, ON, Canada

**Keywords:** Panx1, Panx3, olfaction, ATP, knock out, regulation, compensation

## Abstract

Pannexins (Panx) are integral membrane proteins, with Panx1 being the best-characterized member of the protein family. Panx1 is implicated in sensory processing, and knockout (KO) animal models have become the primary tool to investigate the role(s) of Panx1 in sensory systems. Extending previous work from our group on primary olfaction, the expression patterns of Panxs in the vomeronasal organ (VNO), an auxiliary olfactory sense organ with a role in reproduction and social behavior, were compared. Using qRT-PCR and Immunohistochemistry (IHC), we confirmed the loss of Panx1, found similar Panx2 expression levels in both models, and a significant upregulation of Panx3 in mice with a global ablation of Panx1. Specifically, Panx3 showed upregulated expression in nerve fibers of the non-sensory epithelial layer in juvenile and adult KO mice and in the sensory layer of adults, which overlaps with Panx1 expression areas in WT populations. Since both social behavior and evoked ATP release in the VNO was not compromised in KO animals, we hypothesized that Panx3 could compensate for the loss of Panx1. This led us to compare Panx1 and Panx3 channels *in vitro*, demonstrating similar dye uptake and ATP release properties. Outcomes of this study strongly suggest that Panx3 may functionally compensate for the loss of Panx1 in the VNO of the olfactory system, ensuring sustained chemosensory processing. This finding extends previous reports on the upregulation of Panx3 in arterial walls and the skin of Panx1 KO mice, suggesting that roles of Panx1 warrant uncharacterized safeguarding mechanisms involving Panx3.

## Introduction

The pannexin’s (Panx) is a three-member (in rodents: Panx1, Panx2 and Panx3) family of integral membrane proteins. This gene family has received considerable attention since their initial discovery (Panchin et al., 2000; Bruzzone et al., 2003), with a particular focus on Panx1. The expression of this gene seems ubiquitous throughout the central nervous system (CNS; Ray et al., 2005, 2006) and multiple sensory systems, making Panx1 channels attractive candidates for sensory perception, which is important for interpreting stimuli in an environmental context (Brignall and Cloutier, 2015). Thus far, Panx1 expression in sensory systems has been found in the auditory (Wang et al., 2009), visual (Ray et al., 2005; Dvoriantchikova et al., 2006), gustatory (Huang et al., 2007; Romanov et al., 2007), and main olfactory system (Zhang et al., 2012; Kurtenbach et al., 2014) as well as in pain perception (Zhang et al., 2015, 2017). Given that Panx1 is an adenosine triphosphate (ATP) release channel (Bao et al., 2004), and is present in multiple sensory systems, it is tempting to investigate roles in the modulation of sensory processes.

The introduction of different transgenic mouse lines with distinct Panx1 ablation strategies (Bargiotas et al., 2011; Qu et al., 2011; Dvoriantchikova et al., 2012; Hanstein et al., 2013), has initiated ample opportunities to investigate Panx1 functions in a translational manner—from genes through to systems and behavioral outcomes. The availability of one mouse model (Dvoriantchikova et al., 2012) allowed us to determine a new localization of Panx1 in olfactory sensory neuron (OSN) axon bundles and further dismiss a primary function of Panx1 in olfaction—based upon behavioral testing, electroolfactogram measurements and analysis of ATP release from the olfactory epithelium (OE). The data suggested that Panx1 is one of several alternative pathways to release ATP in the primary olfactory system during chemosensation, likely playing a secondary role only (Kurtenbach et al., 2014).

Here, we report on the presence of Panxs in the accessory olfactory system (AOS), which is home to the accessory olfactory bulb (AOB) and the vomeronasal organ (VNO)—building on previous findings using the same mouse model. The VNO is the primary sensory organ in the AOS. It is located at the base of the nasal septum (Halpern, 1987) and is a chemosensory organ containing specialized sensory neurons called vomeronasal sensory neurons (VSNs), which are found in the pseudostratified neuroepithelium and send signals to the AOB for processing in the brain. In mice, the VSNs recognize chemical signals and initiate innate behavioral responses, like aggressive and reproductive behaviors (Pérez-Gómez et al., 2014). Importantly, ATP appears to play a significant role in triggering these behavioral responses. At the physiological level, ATP release in the VNO is responsible for eliciting a concentration-dependent increase in intracellular calcium, as well as initiating inward currents in VSNs. In turn, increased levels of ATP heighten the responsiveness of VSNs to chemical stimuli (Vick and Delay, 2012). At this time, ATP release is thought to be reliant on purinergic receptor activity upon activation of the vasomotor pump. Exact mechanisms of release are poorly understood, however, known properties of connexins (Cxs) and Panxs (Scemes et al., 2007) suggest that they could contribute either in conjunction or competition with ionotropic P2X or metabotropic P2Y receptors to the release of ATP responsible for signal transduction in the AOS (Gayle and Burnstock, 2005; Vick and Delay, 2012).

To address this knowledge gap, we have characterized the impact of genetic ablation of Panx1 in the AOS. Expression and localization studies demonstrated Panx1 expression in the VNO, with surprising up-regulation of Panx3 both in the sensory and non-sensory neuroepithelium, as well as in nerve fibers innervating the VNO of the Panx1 knockout (KO; ^−/−^) population. Behavioral analysis and a quantitative comparison of steady-state extracellular ATP concentration, via mechanical stimulation in an *ex vivo* preparation, did not reveal significant differences between the two genotypes. The lack of phenotype and the regulation of Panx3 let us conclude that there are likely compensatory mechanisms at play. To further support this conclusion, we showed that in an established cell culture model Panx3 shares some properties with Panx1 including ATP release, as well as responses to stimulation and pharmacological blocking during dye uptake analysis under physiological conditions. To our knowledge, this research provides the first evidence that Panx3 can compensate, at least in part, for Panx1 function(s) in a sensory organ.

## Materials and Methods

### Panx1 Knockout Mice

The generation and initial characterization of Panx1^+/+^ mice (Panx1^fl/fl^) with three LoxP consensus sequences integrated into the Panx1 gene flanking exon 3–4, and KO mice with global loss of Panx1 (Panx1^−/−^, CMV-Cre/Panx1) was described previously (Grundken et al., 2011; Dvoriantchikova et al., 2012; Prochnow et al., 2012). Animals were housed with a 12-h light/dark cycle and had free access to food and water, in compliance to the standards and policies of the Canadian Council on Animal Care (CCAC), and as approved by York University’s standing animal care committee (ACC; protocol 2011-09-GZ). Adult male mice (4–8 months of age) were housed individually 1 week before, and during, behavioral testing.

### Quantitative Real-Time PCR (qRT-PCR)

Total RNA was isolated from adult male mice using the RNAeasy Fibrous Tissue Mini Kit (Invitrogen, Canada), and cDNA was synthesized from 1 μg of total RNA with the ReadyScript cDNA Synthesis Kit (Sigma-Aldrich, Canada), according to the manufacturer’s instructions. qPCR was performed using the SsoFast EvaGreen Supermix (Bio-Rad, Canada) using the following oligonucleotide pairs (Table 1). Experiments were performed in triplicates, using six biological replicates and the CFX Connect^™^ Real-Time PCR Detection System (Bio-Rad, Canada). All experiments included melt curve analysis verifying the identity of PCR amplicons in each reaction. Raw cycle threshold values (Ct-values) were exported from the CFX Manager^™^ Software (Bio-Rad, Canada), and the relative gene expression was calculated using the Relative Expression Software tool (REST; Pfaffl et al., 2002). The REST software reported relative expression values.

**Table 1:** Primer pairs for quantitative Real Time-PCR (qRT-PCR).

Gene	Forward (5′-3′)	Reverse (5′-3′)
mPanx1	CAGGCTGCCTTTGTGGATTC	CGGGCAGGTACAGGAGTATG
mPanx2	GGTACCAAGAAGGCCAAGACT	GGGGTACGGGATTTCCTTCTC
mPanx3	CTTACAACCGTTCCATCCGC	CAGGTACCGCTCTAGCAAGG
P2X7	GACAAACAAAGTCACCCGGAT	CGCTCACCAAAGCAAAGCTAAT
P2X5	TGGAAGGGGTTCGTGTTGTC	AGGGAAGTGTCAATGTCCTGA
P2Y2	CTGGAACCCTGGAATAGCACC	CACACCACGCCATAGGACA
Cx43	ACAGCGGTTGAGTCAGCTTG	GAGAGATGGGGAAGGACTTGT
TRPC2	CTCAAGGGTATGTTGAAGCAGT	GTTGTTTGGGCTTACCACACT
TRPM5	CCTCCGTGCTTTTTGAACTCC	CATAGCCAAAGGTCGTTCCTC
Calhm1	CTGCTGACCACATTACTAGCG	CTGTGCATGTCTCATCGAAGAG
Calhm2	TCTTCAAGAGCAAGGATGTGATG	TCAGTCCATACAGGTAGTTCCG
18s RNA (reference)	TGACTCTTTCGAGGCCCTGTA	TGGAATTACCGCGGCTGCTG
β-Actin (reference)	ATGGAGGGGAATACAGCCC	TTCTTTGCAGCTCCTTCGTT
GAPDH (reference)	TGGATTTGGACGCATTGGTC	TTTGCACTGGTACGTGTTGAT
UBC (reference)	ACCTTTCACTACCTGCGGATG	GTACCCAGGGGCATACTTGC
HSP90 (reference)	TCGTCAGAGCTGATGATGAAGT	GCGTTTAACCCATCCAACTGAAT

### *In Situ* Hybridization (ISH)

Digoxigenin (dig)-labeled sense and antisense riboprobes were prepared from the full-length mPanx1 (NM_019482) coding sequence sub-cloned into the pcDNA3 plasmid (Thermo Fisher, Canada), as described previously (Ray et al., 2005, 2006). After linearization of the plasmid, sense and antisense riboprobes were transcribed using T7 and SP6 RNA polymerase with a digoxigenin RNA labeling mix (Roche, Germany). The ISH was performed as described previously with minor changes (Kurtenbach et al., 2014). Tissue from the VNOs of postnatal day 7 (P7) mice were dissected and immediately embedded in tissue freezing medium (Leica, Germany) at −30°C. Cryostat sections (12 μm) were cut immediately and collected on aminoalkylsilane-treated glass slides. The tissue was subsequently fixed in 4% paraformaldehyde in PBS at 4°C for 20 min, washed in PBS and acetylated by a 15-min treatment in 0.1 M triethanolaminhydrochloride solution with 0.25% acetic anhydride on a stirring plate. Individual sections were rinsed in 2× SSC (30 mM NaCl and 3 mM sodium citrate) and pre-hybridized in hybridization buffer (50% formamide, 5× SSC, 5× Denhardts’ solution, 2.5 mM EDTA, 50 μg/ml heparin, 250 μg/ml tRNA, 500 μg/ml salmon sperm DNA and 0.1% Tween-20) for 1 h at 55°C. Riboprobes were added to the hybridization buffer (0.25 ng/μl), denatured at 80°C for 2 min and applied to tissue sections. Sections were protected from evaporation with cover slips and incubated over night at 55°C in a water-saturated atmosphere. Post hybridization, slides were gently treated with 2× SSC to remove coverslips. Nonspecific binding was removed by wash steps at 55°C with 0.2× SSC for 1 h and then with 0.1× SSC for 15 min. Sections were subsequently equilibrated for 10 min in PBS containing 0.1% Triton X-100 (PBST), blocked with 10% goat serum in PBST buffer for 1 h and then incubated with 1:1000 alkaline phosphatase (AP) conjugated anti-dig Fab fragment (Roche, Germany) in blocking solution over night at 4°C. Subsequently, slides were washed in PBST, equilibrated in B3-Buffer (0.1 Tris-HCl, 0.1 M NaCl, 50 mM MgCl_2_, 0.1% Tween-20) followed by treatment with NBT/BCIP (Roche, Germany) to visualize the hybridization signals.

### Immunohistochemistry (IHC)

Three-week-old and adult mice underwent humane euthanasia, quickly followed by full body perfusion before the separation of the head. The heads were then fixed in 4% PFA at 4°C overnight (ON) and stored until use. After removal of the fur and palate, the VNO was carefully isolated from the bony capsule in physiological Ringers solution (138 mM NaCl, 5 mM KCl, 2 mM CaCl_2_, 2 mM MgCl_2_, 10 mM HEPES, 10 mM Glucose, pH 7.4) and dehydrated in 30% sucrose at 4°C ON before sectioning. Ten micrometer thick cryosections were prepared using a Leica cryostat. Antigen retrieval was completed with 1% SDS for 5 min, followed by three washes each for 5 min with PBS. Sections were blocked with 5% normal goat serum (NGS), 1% bovine serum albumin (BSA), and 0.1% Triton X100 in PBS for 1 h at room temperature (RT). Primary antibodies (polyclonal anti-Panx1 and anti-Panx3 antibodies were kindly provided by Dr. S. Penuela, Western University, ON, Canada, dilution 1:200; G_αo_ SC- 13532, Santa Cruz, CA, USA dilution 1:100; Panx2, 42–2800, Invitrogen, dilution 1:100; NF200 clone NE14, N5389, Sigma-Aldrich, 1:100) were applied in buffer with 1% BSA in PBS containing 0.1% Triton X-100, and incubated at 4°C ON in a humidified atmosphere. After washing in PBS for 30 min, secondary goat anti-rabbit Alexa Fluor 488 and 568 (Invitrogen, Canada) diluted in PBS (1:1000), and applied for 60 min at RT in the dark. After three 10 min washes with PBS, sections were mounted, stained with Fluoroshield^™^ with DAPI (Sigma Aldrich, Canada), sealed, and kept at 4°C ON in the dark. Confocal microscopy was performed using ZEISS LSM 700 microscope, and ZEISS ZEN 2010 software was used to control all imaging parameters. Imaging was performed with 40× or 63× oil, both NA1.4, infinity corrected, DIC objectives. All images were taken using identical settings to allow a direct comparison of tissues from Panx1^+/+^ and Panx1^−/−^ mice. Supplementary Figure S7 shows a secondary antibody control. LSM images were exported into tiff format and assembled using ImageJ and Photoshop CS6.

### Acute* ex Vivo* VNO Preparation for ATP Release Assay

The VNO was carefully dissected as described above. Here, the dissection was carefully conducted in Ringer’s solution (138 mM NaCl, 5 mM KCl, 2 mM CaCl_2_, 2 mM MgCl_2_, 10 mM HEPES, 10 mM Glucose, pH 7.4) under an Olympus SZ61 dissecting microscope to ensure minimal mechanical stimulation or damage to the structure. Isolated VNOs were equilibrated in fresh Ringers solution for 45 min at RT. The Ringers solution was replaced before stimulation and non-stimulation control conditions. Non-stimulated controls remained at rest in Eppendorf tubes for 10-min. Mechanical stimulation was induced by carefully pipetting up and down the Ringers solution around the VNO for a consecutive 10-min period, under visual control ensuring not to capture or disrupt the VNO. After the 10-min incubation time, 50 μl aliquots of the supernatant were removed, heated for 1 min at 95°C, and stored on ice for detecting ATP concentrations (see section below titled “*In vitro* Luciferase Assay for ATP Determination”). All experiments were repeated five times for ATP measured from the VNO of either genotype.

### Behavioral Test: Modified Resident-Intruder Assay

Male mice (4–8 months) were housed individually for 1 week before testing. Before the test, the bedding of the resident mice (same age) cages was not changed for three days. Mice were placed into home cages of other male mice for 10 min, which were covered with a glass plate for the duration of the test. Behaviors were recorded on video, and the animals’ interactions were quantified manually by counting and timing the specific behaviors. Biting, tail rattling/flicking, chasing, cornering and tumbling were considered aggressive behaviors, which were quantified using different parameters such as attack and defensive posture frequency and latency. Smell time was also used as a parameter of the investigation. To test for statistical significance student’s *T*-test was used.

### Plasmid Constructs

Expression vectors contained the full-length open reading frames of mPanx1 (NM_019482, amino acids (aa) 1–426) and mPanx3 (NM_172454, aa 1–392) were cloned into the pEGFP-N1 expression vector (Clontech Laboratories Inc., Mountain View, CA, USA) in two steps. First, the open reading frames were synthesized as gBlocks (Integrated DNA Technologies Inc. (IDT), Coralville, IA, USA) and cloned into the TA cloning vector pJet1.2 (Thermo Fisher Inc., Mississauga, ON, Canada). Then the coding regions were isolated by restriction digest and cloned in-frame into the pEGFP-N1 expression vector. All plasmid constructs used in this study were sequence verified (Eurofins MWG Operon LLC, Huntsville, AL, USA).

### Cell Culture, Transient Transfection, Western Blot and Confocal Imaging

Neuroblastoma 2a (Neuro2a) cells (Olmsted et al., 1970) were cultivated in DMEM with 2 mM glutamine, 1% non-essential amino acids (NEAA), 1% penicillin and streptomycin (PS) and 10% fetal bovine serum (FBS) at 37°C in a humidified atmosphere with 5% CO_2_. Approximately 30,000 cells were seeded in each well of 24-well plates or glass-bottom dishes (MatTek Corporation, Ashland, MA, USA) and transfected with 400 ng endotoxin-free plasmid DNA, using the Effectene transfection protocol (Qiagen Inc., Valencia, CA, USA). For western blot, whole cell protein lysates were prepared 48 h after transfection. Twenty microgram protein was separated by 10% SDS-PAGE, transferred to 0.2 μm Midi format nitrocellulose membrane and processed using the iBind^™^ Western System (Bio-Rad Inc., Mississauga, ON, Canada). Primary antibodies were diluted 1:1000 (mouse anti-GFP, Roche; rabbit anti-GFP (FL), Santa Cruz Biotechnologies, TX, USA) and 1:20,000 (mouse anti-β-actin; Sigma-Aldrich Chemie GmbH, Munich, Germany). The secondary antibodies (LI-COR Biosciences, St. Lincoln, NE, USA) were diluted 1:20,000 (donkey anti-rabbit IRDye680LT) or 1:20,000 (goat anti-mouse IRDye800CW). Signals were detected using the Odyssey^®^ CLx Infrared Imaging System (LI-COR Biosciences).

For confocal microscopy, transfected cells were fixed with 4% paraformaldehyde for 20 min at RT, washed with PBS, and mounted with Fluoroshield^™^ with DAPI (Sigma Aldrich, Canada) for imaging. Samples were visualized using a Zeiss LSM 700 confocal microscope with a Plan-Apochromat 63×/1.4 Oil DIC M27 objective and the ZEN 2010 program to control all hardware parameters. Images were collected by line averaging (4×) at high resolution (2048 × 2048 pixel) using single planes. Images were exported and further processed using ImageJ and finally were combined using Adobe Photoshop CS6 for presentation.

### Fluorescent Dye Uptake Assay

This assay is a variation of the ethidium bromide uptake assay previously reported (Prochnow et al., 2009; Kurtenbach et al., 2013). Neuro2A cells were grown in 3.5 cm MatTek cell culture dishes and transiently transfected with 400 ng EYFP, EGFP, mPanx1-EYFP or mPanx3-EGFP as described above. After 48 h, cultures were equilibrated for 30 min at 37°C and 5% CO_2_ in 1 mL complete DMEM with 2 mM glutamine, 1% NEAA, 1% penicillin and streptomycin (PS) and 10% FBS, but lacking phenol red (Supplementary Figure S4). MatTek chambers were placed in a live cell imaging chamber attached to the sample stage using a Zeiss 700 confocal microscope. Cells expressing mPanx1 and mPanx3 were selected for imaging at 37°C. Then, 1 mL fresh complete DMEM was added to cultures, to reach a final concentration of 10 μM ethidium bromide (EtBr). Treatment conditions included the application of 50 mM KGlu, 140 mM KGlu, or 3 μM ATP, as well as Panx1 blockers (3 mM ATP, 500 μM probenecid, 10 μM BB FCF). For blocking, cultures incubated for 5 min with blocker alone before application of 140 mMKGlu and EtBr. Images were taken at 1-min intervals, and dye uptake was measured over a period of 20 min.

Normalized dye uptake values were calculated by the change in fluorescence of the red channel (EtBr uptake) over 20 min and the protein expression documented by the fluorescence of the green channel (EGFP/EYFP fluorescence). All experiments were repeated at least three times. An automated cell selection and analysis of fluorescence protocol was created using a collection of plugins built into the ImageJ software. At the start, separated channels were background subtracted. A morphological segmentation tool from the MorphoLibJ plugin library was applied to segment images based on the watershed algorithm, creating new images based on watershed lines. These images were inverted to analyze particles, and the resulting regions of interest (see Supplementary Figure S5 as an example) were filtered using the HiLo algorithm to exclude saturated regions in images. Next, integrated density values were measured from the regions of interest within the green and red channels. As there is a linear relationship between protein expression (green channel) and the amount of dye uptake (red channel; see Supplementary Figure S3), it was possible to normalize dye uptake by calculating the ratio of dye uptake (in 20 min) and the amount of protein fluorescence on a cell to cell basis. Statistical analysis was completed using the Wilcoxin Mann Whitney *U* test.

### *In Vitro* Luciferase Assay for ATP Determination

ATP assays were performed in a 96 well format (Greiner Bio-One, Canada) using the Molecular Probes^®^ ATP Determination Kit as described by the manufacturer (Life Technologies, USA). Each well was seeded with 10,000 transiently transfected Neuro2a. Samples were measured using the Synergy H4 hybrid multi-well plate reader (Biotek, USA) as reported previously (Kurtenbach et al., 2014). ATP concentrations in experimental samples were determined from ATP standard curves (concentrations: 0 μM, 1 μM, 5 μM, 10 μM and 25 μM), dissolved in 1× TE buffer, included in each assay. The Gen5 Data Analysis Software (BioTek) was used to set luminescent assay parameters, including automatic gain settings and 5 s integration time per well, and complete data exportation. The data analysis was completed in MS-Excel, and the student’s *T*-test was used to test for statistical significance. All *in vitro* ATP assays were repeated three times. See Supplementary Figure S6 for a representation of luciferase assay methodology *in vitro*.

### Blocker Pharmacology and Stimulants

Simulants included 3 μM adenosine triphosphate (ATP, pH-buffered, Sigma-Aldrich), 50 mM and 140 mM potassium gluconate (KGlu, Sigma-Aldrich). Blockers used include; 50 μM carbenoxolone (CBX, Sigma-Aldrich), 100 nM mefloquine (MFQ, QU024-1, BioBlocks), 10 μM brilliant blue food dye (BB FCF, Sigma Aldrich). It is important to note that all blocking data were collected after incubation for 5 min before application of 140 mM KGlu and EtBr, and recordings were taken starting upon the application of EtBr.

### Statistical Analysis

Statistical analysis and data presentation were performed using Mathworks Matlab software or Microsoft Excel. Experiments were repeated at least three times. In most experiments, at least three independent replicates were used. All data were analyzed for data distribution and subjected to Mann-Whitney *U* tests for independent samples or a paired *t*-test, when appropriate. Figures were then compiled in Adobe Illustrator CC 2015 or Photoshop CS5 or higher.

## Results

### Pannexin3 mRNA Expression Is Upregulated in the Vomeronasal Organ of Panx1^−/−^ Mice

**Figure 1:**
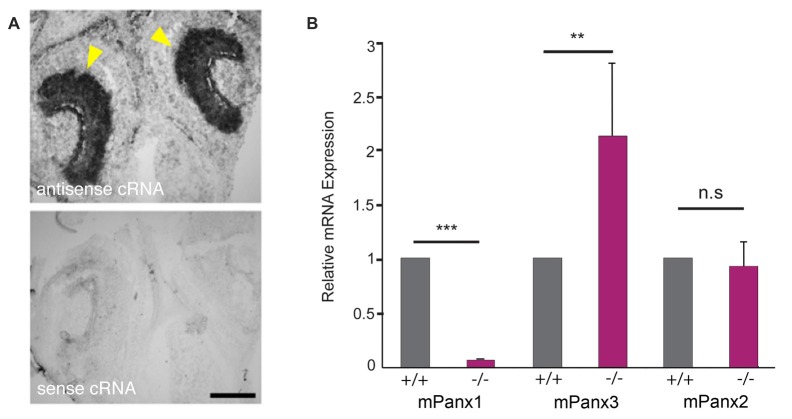
Pannexin mRNA expression in the mouse VNO. **(A)** *In situ* hybridizations (ISHs) from juvenile mice (P7) with mPanx1 riboprobes. Panx1 antisense cRNA delivered strong labeling in the VNO. The strongest staining was detectable in the basal cell layer of the sensory epithelium (SE) indicated by yellow arrowheads. Note the absence of specific staining when the Panx1 sense cRNA was used. VNO = vomeronasal organ. Scale bar = 100 μm. **(B)** Quantitative room temperature (RT)-PCR data for Panx expression in the VNO. Panx1^−/−^ mice lacked detectable mPanx1 expression in the VNO (*n* = 6). Mouse Panx3 mRNA expression showed a significant 2-fold upregulation in the VNO of Panx1^−/−^ mice. No differential mRNA regulation was found for mPanx2. Primers specific for 18S rRNA were used as a reference. Experiments were performed in triplicates; significance is denoted by asterisks: Student’s *T*-test ***p < 0.001, **p < 0.01, Error Bars denote SEM.

*In situ* hybridizations with cRNA probes specific for mouse Panx1 (mPanx1; Ray et al., 2005) confirmed expression of mPanx1 in the vomeronasal organ (VNO; Figure 1A). The antisense cRNA revealed strong staining of the VNO epithelia of 7-day-old mice, indicated by the yellow arrowheads, while the sense RNA probes only generated very faint staining, verifying the probes’ specificity. Next, Panx expression was quantified in total RNA isolated from the VNO, using quantitative Real Time-PCR (qRT-PCR) and primers specific for the mPanx1 (GI:8626133), mPanx2 (GI:163838634) and mPanx3 (GI:86262154) mRNAs. Significant expression of mPanx1 was detected in the VNO of adult wild-type mice (Figure 1B). Consistent with the gene targeting strategy ablating exons 3 and 4 of the Panx1 gene (Dvoriantchikova et al., 2012), no mPanx1 expression was detected in the VNO of mPanx1^−/−^ animals (relative expression ratio: 0.0019 ± 0.0006 fold, *p* < 0.0001, *n* = 6; mean cycle threshold (Ct) ± SD: wild type 30.36 ± 0.87, mPanx1^−/−^ 38.56 ± 1.61). mPanx2 and mPanx3 mRNA expression were determined to address the possibility of compensatory mechanisms that may affect Panx expression. A significant upregulation of the mPanx3 mRNA expression (2.03 ± 0.78 fold, *p* = 0.007, *n* = 6; Ct ± SD: wild type 30.47 ± 1.01, mPanx1^−/−^ 29.50 ± 0.39) in the VNO of Panx1^−/−^ mice was measured. In contrast, no change in the expression of mPanx2 was found in the VNO of the Panx1^−/−^ mice (1.08 ± 0.23 fold, *p* = 0.89, *n* = 6, Ct ± SD: wild type 30.09 ± 0.21, mPanx1^−/−^ 30.24 ± 0.46).

### Localization of Pannexin Proteins in the VNO of Wild-Type and Panx1^−/−^ Mice

**Figure 2:**
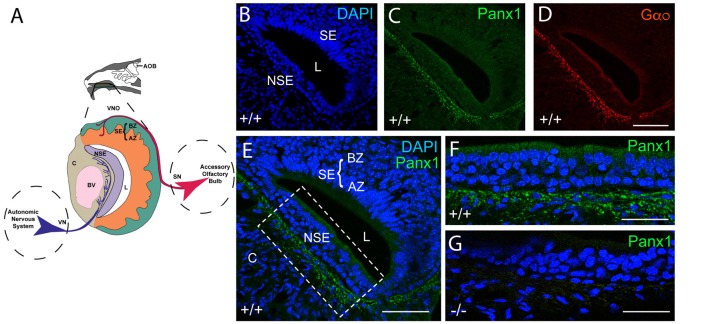
Panx1 protein expression in the NSE of the juvenile mouse VNO. **(A)** Overview of the murine VNO adapted from Sánchez-Andrade and Logan (2014) **(B–D)**. The intact wild-type VNO imaged at 40× magnification is shown in **(B)** depicting DAPI staining of nuclei, the localization of **(C)** mPanx1 and **(D)** of the G-alpha protein Gαo. The region highlighted by the box in the overview in **(E)** is shown at 63× magnification in **(F)** highlighting the significant mPanx1 expression in the basal and lower expression in the ciliated epithelium of the NSE. **(G)** A comparable region in Panx1^−/−^ mice lacked Panx1 expression. Abbreviations: AZ, apical zone; BZ, basal zone; SE, sensory epithelium; L, lumen; NSE, non-sensory epithelium; C, cavernous tissue; BV, a blood vessel. Scale bars: **(B–E)** = 100 μm, **(F,G)** = 50 μm.

The mouse VNO is part of the AOS, composed of sensory and non-sensory layers as shown in the cartoon in Figure 2A. In the sensory layer, cell bodies of the sensory neurons, situated in the sensory epithelium (SE), segregate into the apical and basal zones (AZ, BZ) and project to the anterior or posterior portion of the AOB (details not shown) respectively. Cells in the non-sensory layer (NSE) are separated by a nerve fiber tract responsible for the autonomous innervation of local assemblies of the vasculature in cavernous tissue, which respond to increased sympathetic tone with vasoconstriction and in turn regulate the lumen (L) of the VNO.

The localization of Panx proteins in the VNO of juvenile mice was tested by IHC. Abundant mPanx1 protein expression was found in nerve fibers innervating blood vessels in the cavernous part of the VNO, with relatively lower levels of expression in the non-sensory epithelial layers (Figures 2B,C). Further, the Gαo protein, a protein known to couple neurotransmitter receptors to ion channels in sympathetic neurons (Jeong and Ikeda, 1998), co-localized with mPanx1 immunoreactive nerve fibers (Figure 2D). The overview in Figure 2E shows that expression of mPanx1 in the sensory part of the VNO was neglegible. Zoomed in views of the complementary areas of the non-sensory part of the VNO using identical immunostaining and imaging conditions, demonstrated an absence of mPanx1 immunoreactivity in Panx1^−/−^ mice (Figures 2F,G).

**Figure 3:**
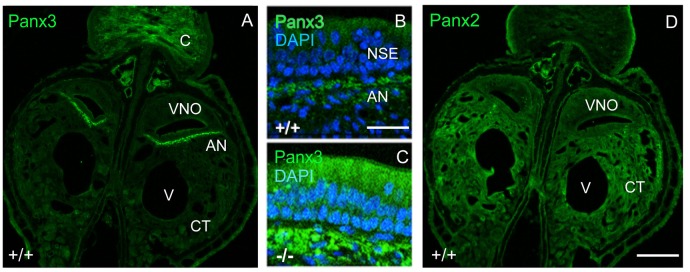
Localization of mPanx2 and mPanx3 proteins showing upregulation of mPanx3 in the NSE of juvenile Panx1^−/−^ mice. **(A)** The left panel demonstrates an overview taken at 10x magnification showing the entire VNO and surrounding structures after Panx3 immunostaining. Mouse Panx3 has increased expression where mPanx1 has been demonstrated previously to be found, basal to the NSE, in the autonomous sensory nerve. There is also increased expression in the cartilaginous zone, which is characteristic of mPanx3. **(B,C)** The two panels in the middle show higher magnifications (63×) of the region indicated by the white box, demonstrating some mPanx3 expression in the cilia of the NSE and cell bodies basal to the NSE in wild-type mice. **(C)** The panel below shows that there is a clear increase in mPanx3 expression in Panx1^−/−^ in the same regions. **(D)** Mouse Panx2 is ubiquitously localized in the VNO and the CT. Abbreviations: VNO, vomeronasal organ; V, vasculature; CT, cavernous tissue; C, cartilage; NSE, non-sensory epithelium; AN, autonomous (parasympathetic/sympathetic) nerve; +/+ = WT, −/− = Panx1 knockout (KO). Scale bars 10× overviews = 350 μm, 63× magnifications = 50 μm.

Interestingly, in juvenile wild-type mice, mPanx3 expression was detectable and confined to regions similar to mPanx1, as well as in cartilage (Figure 3A). Meanwhile, in Panx1^−/−^ mice, Panx3 immunoreactivity was elevated in the non-sensory part of the VNO in regions of Panx1 protein expression in wild-type controls, but not detectable in the SE (Figure 3). This result strongly argues for protein upregulation in subpopulations of cells with loss of mPanx1 expression or nearby thereof. In contrast, mPanx2 was equally expressed in wild-type and Panx1^−/−^ populations, demonstrating ubiquitous expression within and beyond the VNO (Figure 3), and showing higher levels of expression in connective tissue (CT) compared to mPanx1 and mPanx3. The significant expression of the Panx2 protein in the VNO, despite low mRNA levels, is consistent with a previous report demonstrating that Panx1, 2 and 3 mRNA and protein expression were disconnected in most tissues (Le Vasseur et al., 2014).

**Figure 4:**
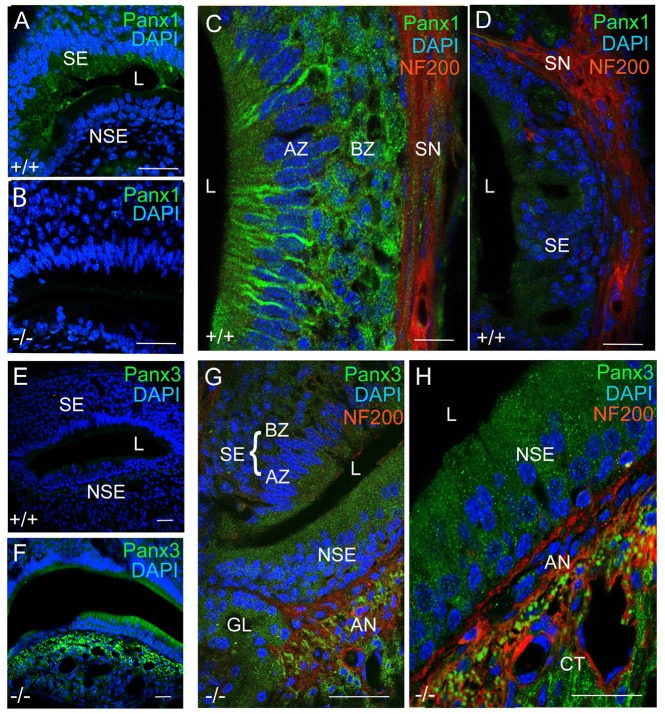
Panx1 and Panx3 protein localization in the VNO of adult mice. **(A,B)** Overviews showing mPanx1 expression (green) and DAPI staining (blue) in the VNO of wild-type and Panx1^−/−^ mice. In the adult Panx1, wild-type VNO the mPanx1 protein is detected in the SE layer, with some expression in the NSE layer. The Panx1^−/−^ VNO showed no mPanx1 staining. Scale bars **(A,B)** = 200 μm. **(C)** In the SE layer of wild-type mice, the mPanx1 protein (green) is localized along tracts of sensory neurons in both AZ and BZ, with some immunoreactivity found in NF200 (red) immunoreactive nerve fibers (SN). The mPanx1 protein showed punctate localization in both the apical zone (AZ) and basal zone (BZ). A very low level of mPanx3 immunoreactivity was detectable in the SE layer, which was dismissed as background **(D)** Scale bars **(C,D)** = 50 μm. **(E,F)** Overviews include DAPI staining of nuclei (blue) and Panx3 expression (green) in wild-type and Panx1^−/−^ mice, showing upregulation of Panx3 expression in the VNO of Panx1^−/−^ mice. Scale bars **(E,F)** = 100 μm. At higher magnification **(G,H)** of VNOs from Panx1^−/−^ mice showed localization of Panx3 in both the SE and NSE layers, and the NF200 immunoreactive nerve fibers, with the highest magnification demonstrating a punctate pattern of localization. Scale bars **(G,H)** = 100 μm, 50 μm. Abbreviations: SE, sensory epithelium; NSE, non-sensory epithelium; AZ, apical zone; BZ, basal Zone; L, lumen; SN, sensory nerve; AN, autonomous (parasympathetic/sympathetic) nerve; GL, gland.

Next, we investigated mPanx1 and mPanx3 protein localization in the VNO of adult mice. Abundant mPanx1 expression was detected in the sensory epithelial (SE) layer, with negligible levels of expression in the non-sensory epithelial (NSE) layer. Meanwhile, no mPanx1 protein expression was found in Panx1^−/−^ mice (Figures 4A,B). At higher magnification, images of the SE of wild-type animals revealed Panx1 localization along the tracts of neurons in the AZ as well as distinct punctate expression in the BZ (Figure 4C). In the SE of the VNO of wild-type mice, only traces of the mPanx3 protein were found, which we dismissed as background staining (Figures 4D,E). However, similar to juvenile Panx1^−/−^ mice, the mPanx3 protein was significantly upregulated in both the SE and NSE (Figure 4F). The zoomed in views shown in Figures 4G,H revealed that mPanx3 was localized in both layers of the SE, as well as in the NSE, AN and the cavernous tissue. At the highest magnification, mPanx3 showed a punctate localization. Together, the tissue distribution of mPanx1 protein found in adult and P7 mice tissue partially overlapped with the mPanx3 protein expression found in Panx1^−/−^ mice, raising the question of whether mPanx3 could functionally compensate for the loss of mPanx1.

### ATP Release in the VNO of Wild-Type and Panx1^−/−^ Mice

**Figure 5:**
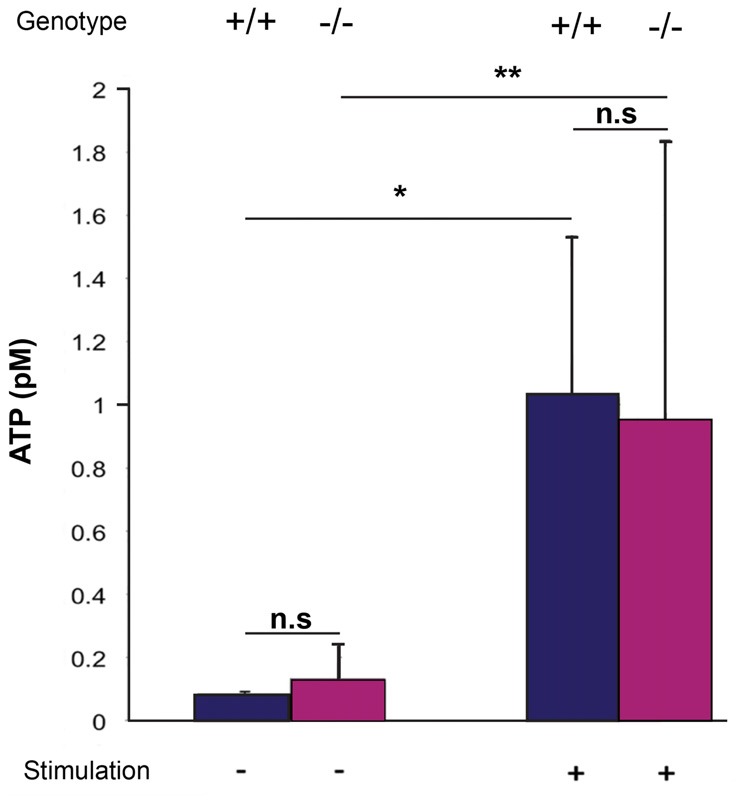
Quantification of extracellular ATP after mechanical stimulation of the VNO. ATP release from the VNO of wild-type and knock out animals was determined as outlined in materials and methods using a luminescent ATP detection assays. Without stimulations, very low levels of ATP were detectable, with no differences between the two genotypes. Mechanical stimulation significantly increased ATP release, again, with no difference between wild-type and Panx1^−/−^ animals. Error bars indicate standard deviation. *p < 0.01, **p < 0.05.

ATP is a known mediator of VSNs, and Panx1 has been described as a major ATP release channel. Since ATP release can be evoked in the adult VNO by mechanical stimulation (Vick and Delay, 2012), it is possible to quantify extracellular ATP using acute *ex vivo* preparations of the VNO. Here, basal levels of extracellular ATP were detectable in the VNO of both adult Panx1^+/+^ and Panx1^−/−^ mice (ATP concentration: Panx1^+/+^, 0.08 ± 0.007 pM, Panx1^−/−^, 0.13 ± 0.1 pM; *p* = 0.5; *n* = 5). Repeated mechanical stimulations reliably elevated ATP release in both Panx1^+/+^ and Panx1^−/−^ mice VNO preparations (Panx1^+/+^, 1.02 ± 0.5 pM; Panx1^−/−^, 0.95 ± 0.9 pM, *p* = 0.9; *n* = 5; Figure 5). The increase was significant for both genotypes (Panx1^+/+^, *p* = 0.01; Panx1^−/−^, *p* = 0.05), although the concentration of ATP was indistinguishable for both non-stimulated and stimulated conditions. Since evoked ATP release in the VNO was not compromised in KO animals, differential expression of genes encoding for proteins known for interacting with ATP (purinergic receptors), or channels permeable for ATP (connexin, calcium homeostasis modulator), or opening upon mechanical stimulation (transient receptor potential cation channel) were investigated. Using qPCR, the expression of connexin-43 (Cx43), the transient receptor potential cation channel, subfamily C, gene 2 (TRPC2), the transient receptor potential cation channel, subfamily M, gene 5 (TRPM5), the purinergic receptors P2X7, P2X5 and P2Y2, as well as the calcium homeostasis modulator 1 and 2 (Calhm1, Calhm2) were detectable in the VNO. Non-significant expression changes were found for the purinergic receptors P2X7 (Mean ± Standard Error; 0.893 ± 0.310, *p* = 0.541) and P2X5 (1.235 ± 0.354, *p* = 0.250), as well as TRPC2 (1.351 ± 0.450, *p* = 0.138). Furthermore, Cx43 (Cx43: 0.397 ± 0.144, *p* = 0.004), P2Y2 (0.533 ± 0.183, *p* = 0.0001), TRPM5 (0.506 ± 0.201, *p* = 0.016), Calhm1 (0.369 ± 0.176, *p* = 0.011) and Calhm2 (0.477 ± 0.166, *p* = 0.001) were significantly downregulated. Together, evidence for compensatory upregulation of the most likely alternative sources contributing to ATP release in the VNO was not found. This result suggests that compensatory upregulation of Panx3, and ATP release through Panx3, could contribute to the similar increase in ATP release upon mechanical stimulation in both genotypes.

### Aggression and Defensive Behavior in Wild-Type and Panx1^−/−^ Mice

**Figure 6:**
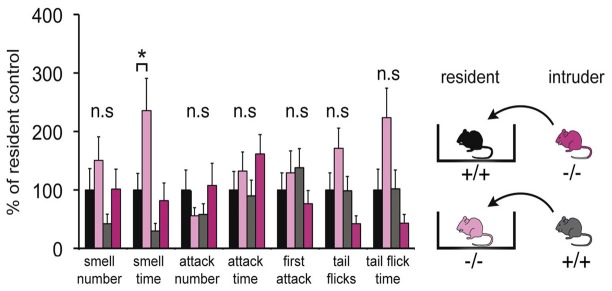
*Behavioral comparison of Panx1^+/+^ and Panx1^−/−^ mice*. This modified intruder assay with *N* = 14 (Panx1^+/+^) and *N* = 15 (Panx1^−/−^) mice demonstrates that there are no significant differences in three behavioral categories, including aggressive behaviors mediated by the VNO (tail flicks, attacks indicated by biting, chasing, cornering and tumbling), between the two different mouse lines. A significant increase in smell time was found for Panx1 KO resident mice. The cartoon outlines the strategy used. Error bars indicate SEM. *p < 0.05.

Since the VNO plays a major role in social interactions, a modified resident-intruder assay was performed to identify discernible behavioral differences between the two populations. Adult male mice were used to test for aggressive and defensive behaviors by the resident, as well as social stress evoked by the intruder. This analysis included quantification of three categories, smell, attack and tail flicks, which are typical behaviors elicited by specific chemosensory ligands present in the sensory epithelial layer of the VNO. Figure 6 shows that adult Panx1^−/−^ mice (*n* = 14) did not show any significant difference in these measures compared to age-matched wild-type Panx1 (^+/+^) mice (*n* = 15). Interestingly, no significant differences were found in six behavioral responses. A single significant increase in smell time of Panx1 KO animals was observed when intruders were placed in their cage. In summary, the behavioral data suggested that lack of Panx1 function(s) in the VNO were not affecting behavioral outcomes in the knock out mice. Considering that the change in behavior was minor, albeit statistically significant, the result was interpreted as an indication of a compensatory role of Panx3.

### Expression Properties of Panx1 and Panx3 in Neuro2a Cells Are Similar

**Figure 7:**
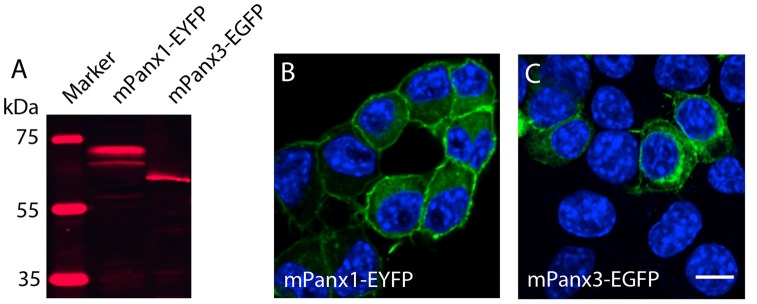
Panx1 and Panx3 localization in Neuro2A cells. **(A)** Western blot showing Panx1-EYFP and Panx3-EGFP proteins 48 h after transient transfection into Neuro2A cells. **(B,C)** Merged images, including DAPI staining of nuclei (blue), and Panx1-EYFP (Left) and Panx3-EGFP (Right) localization (green) in Neuro2a cells. Scale bars = 10 μm.

Testing the hypothesis that mPanx3 could functionally compensate for mPanx1 in Panx1^−/−^ mice, we analyzed both proteins in Neuro2a cells. It is worth noting that Neuro2a cells express Panx1 mRNA, but not Panx2 or Panx3 (Supplementary Figure S1), however, very low levels of endogenous Panx1 protein expression have not been detected by western blot (data not shown). Transient transfection of mPanx1-EYFP and mPanx3-EGFP increased steady-state mRNAs ≈95 fold (mPanx1) or ≈3400 fold (mPanx3) relative to endogenous levels (Supplementary Figure S2). The western blot analysis after transient overexpression of mPanx1-EYFP and mPanx3-EGFP in Neuro2a cells demonstrated that both proteins were expressed similarly to previous reports (Boassa et al., 2007; Penuela et al., 2007, 2008; Figure 7A). Also, confocal imaging revealed that both proteins localized in the cell membrane and internal membranes, consistent with the typical distribution of Panx proteins (Figures 7B,C).

### Mouse Panx1 and Panx3 Release ATP *in Vitro*

**Figure 8:**
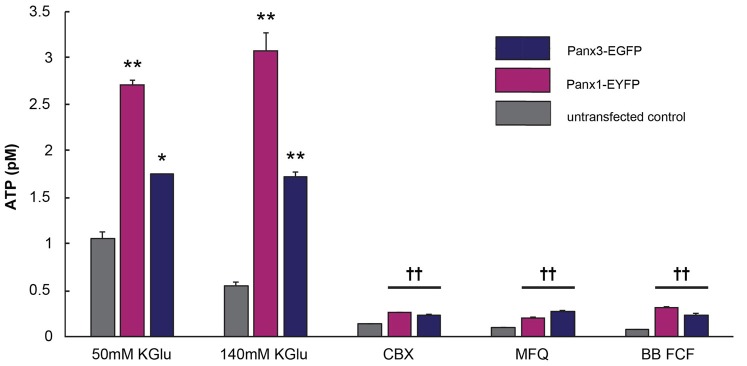
Panx3 has ATP releasing properties like Panx1. Luciferase assay depicting the amount of released ATP by Neuro2A cells that were untransfected (gray) or transfected with mPanx1 (Pink) or mPanx3 (Indigo). Using either medium (50 mM) or high (140 mM) concentrations of potassium gluconate (KGlu) stimulates mPanx1 and mPanx3 to release significantly more ATP compared to Neuro2A cells. Traditional Panx1 blockers carbenoxolone (CBX), mefloquine (MFQ) and brilliant blue food dye (BB FCF) also block mPanx3 and Neuro2a cells when stimulated with 140 mM KGlu prior incubation with the blockers. *Indicate significance compared to Neuro2A controls, *p < 0.001, **p < 0001. ^††^Indicates significance compared to 140 mM KGlu stimulation condition. ^††^*p* < 0.001.

Panx1 channels are well established ATP release channels and the expression and localization of mPanx1 and mPanx3 were similar *in vitro*, therefore, an *in vitro* luciferase assay was used to test whether Panx3 demonstrated similar ATP release channel properties. As shown in Figure 8, both mPanx1-EYFP and mPanx3-EGFP release detectable amounts of ATP upon stimulation with 50 mM potassium gluconate (ATP concentration in pM: mPanx1, 50 mM KGlu: 2.71 ± 0.05, *p* value = 5.06e^−5^; mPanx3: 50 mM KGlu 1.75 ± 0.006, *p* value = 7.10e^−4^, *N* = 3 independent experiments) and 140 mM KGlu (mPanx1: 140 mM KGlu 3.09 ± 0.19, *p* value = 1.78e^−4^; mPanx3: 140 mM KGlu 1.72 ± 0.05, *p* value = 4.55e^−5^, *N* = 3 independent experiments), when normalized to EGFP or EYFP transfected Neuro2a cells with no stimulation, as well as when compared to non-transfected Neuro2A cells under the same conditions (Neuro2A: 50 mM KGlu 1.05 ± 0.07, *N* = 3; 140 mM KGlu 0.55 ± 0.3, *N* = 3). The specificity of the observed ATP release was tested using the Panx1 blockers carbenoxelone (CBX), mefloquine (MFQ) and blue food dye (BB FCF). All blockers showed reliable reduction of ATP release induced by high potassium when cells expressed mPanx1 (CBX 0.26 ± 0.005, *p* value = 1.10e^−4^, *N* = 3; MFQ 0.20 ± 0.007, *p* value = 1.10e^−4^, *N* = 3; BB FCF 0.31 ± 0.01, *p* value = 1.20e^−4^, *N* = 3). Interestingly, all Panx1 blockers caused the same effect on Panx3 expressing cultures (CBX 0.23 ± 0.01, *p* value = 8.71e^−6^, *N* = 3; MFQ 0.27 ± 0.01, *p* value = 1.05e^−5^, *N* = 3; BB FCF 0.23 ± 0.02, *p* value = 1.05e^−5^, *N* = 3). It is important to note that these blockers also reduce the amount of ATP release in non-transfected Neuro2A cells as well (CBX 0.14 ± 0.003, *p* value = 3.00e^−4^, *N* = 3; MFQ 0.10 ± 0.002, *p* value = 2.06e^−4^, *N* = 3; BB FCF 0.07 ± 0.005, *p* value = 1.77e^−4^, *N* = 3), an observation we attribute to endogenously expressed low levels of mPanx1. These results established similar channel properties *in vitro*, with respect to ATP release, amongst mPanx1 and mPanx3 in response to medium and high concentrations of KGlu and inhibitors that act upon mPanx1. This provides a strong rationale to continue comparing channel function properties *in vitro* and investigation the potential functional replacement of Panx1.

### Dye Uptake Properties of Panx1 and Panx3 in Neuro2a Cells Are Similar

**Figure 9:**
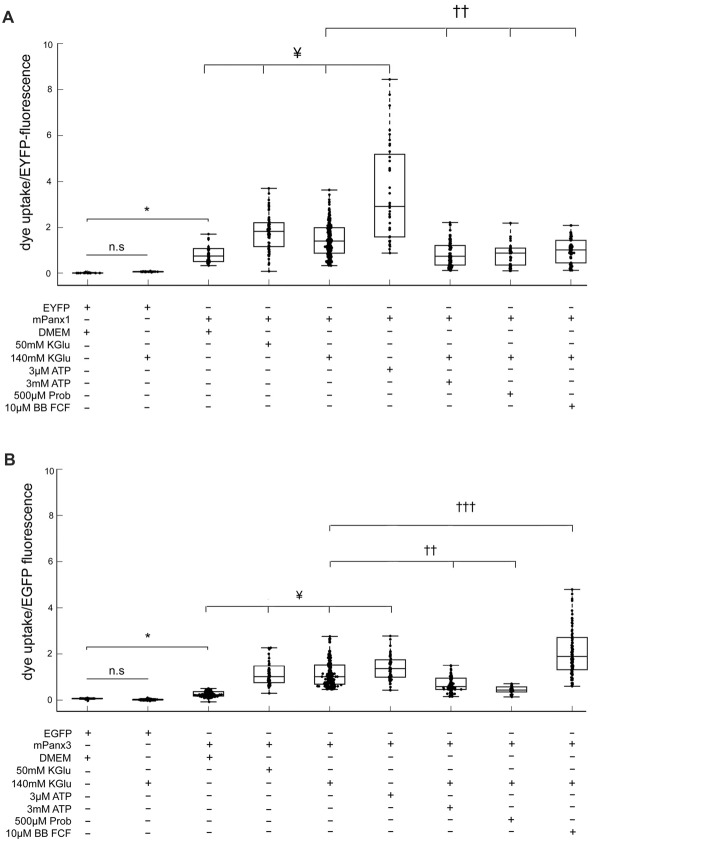
Panx1 and Panx3 dye uptake properties. EtBr uptake assay is depicting the amount of dye taken up by Neuro2A cells that were transfected with EYFP, EGFP, mPanx1-EYFP or with mPanx3-EGFP. **(A)** Neuro2A-EYFP cells did not uptake dye, even when trying to stimulate with 140 mMKGlu. However, using either 50 mM KGlu, 140 mM KGlu, 3 μM ATP on mPanx1 transfected cultures, significantly stimulated channel opening. 3 mM ATP, 500 μM Prob or 10 μM BB FCF application, after stimulation with 140 mM KGlu for 5 min, block the channel from taking up the dye. **(B)** EtBr uptake assay is depicting the amount of dye taken up by Neuro2A cells that were transfected with EGFP or with mPanx3. Neuro2A-EGFP cells did not uptake dye, even when trying to stimulate with 140 mMKGlu. However, using either 50 mM KGlu, 140 mM KGlu, 3 μM ATP on mPanx3 transfected cultures, significantly stimulated channel opening. 3 mM ATP or 500 μM Prob application, after stimulation with 140 mM KGlu for 5 min, block the channel from taking up the dye. Treatment with 10 μM BB FCF after application of 140 mMKGlu enhanced dye uptake activity significantly compared to treatment with 140 mMKGlu alone. *Indicate significance compared to Neuro2A controls, *p < 0.0001. ^¥^Indicate significance compared to basal conditions, ^¥^*p* < 0.0001. ^††^Indicates significant decrease compared to 140 mM KGlu stimulation conditions, ^†††^indicates significant increase compared to 140 mM KGlu stimulation conditions ^††^*p* < 0.0001 and ^†††^*p* < 0.0001. KGlu (potassium gluconate), ATP (adenosine triphosphate), Prob (probenecid), BB FCF (Brilliant blue food dye).

ATP release properties of mPanx1 and mPanx3 were comparable *in vitro*. Hence, a robust ethidium bromide (EtBr) uptake assay (Kurtenbach et al., 2013; Shao et al., 2016) was used to further test channel activities. In this assay (Figures 9A,B), Neuro2a cells expressing either EYFP or EGFP showed no dye uptake under physiological conditions in complete growth medium (EYFP: 0.026 ± 6.87e^−4^, EGFP: 0.060 ± 2.30e^−3^), as well as after stimulation with 140 mM KGlu (EYFP: 0.078 ± 8.50e^−4^, EGFP: 0.022 ± 1.20e^−3^). In contrast, Neuro2a cells overexpressing mPanx1-EYFP or Panx3-EGFP showed an increasing and linear relationship between the amount of dye uptake and protein expression under both physiological conditions (mPanx1: *R*^2^ = 0.89, mPanx3: *R*^2^ = 0.90; Supplementary Figure S3A) and after treatment with 140 mM KGlu (mPanx1: *R*^2^ = 0.88, mPanx3: *R*^2^ = 0.91; Supplementary Figure S3B), demonstrating that the dye uptake was correlated with Panx expression. Therefore, dye uptake analysis was conducted after normalizing the amount of dye uptake to the level of protein expression.

Mouse Panx1-EYFP expression caused significantly increased dye uptake under basal conditions (0.9544 ± 0.018, *n* = 198, *p* value = 4.46e^−10^) when compared to EYFP transfected cells (Figure 9A). In addition, treatment with 50 mM KGlu (1.78 ± 0.012, *n* = 114, *p* value = 2.95e^−8^), 140 mM KGlu (1.51 ± 0.0042, *n* = 407, *p* value = 2.45e^−6^), or 3 μM ATP (3.63 ± 0.061, *n* = 90, *p* value = 1.92e^−11^) caused a significant increase in dye uptake when compared to mPanx1-EYFP under physiological conditions. In contrast, treatment with 3 mM ATP (0.87 ± 0.0067, *n* = 125, *p* value = 1.85e^−10^), 500 μM probenecid (Prob; 0.85 ± 0.013, *n* = 407, *p* value = 3.88e^−7^) or 10 μM brilliant blue food dye (BB FCF; 0.99 ± 6.6e^−3^, *n* = 407, *p* value = 1.59e^−6^) prior to stimulation with 140 mMKGlu, a condition previously reported to open Panx1 channels from different species and in difference expression systems showed a significant decrease of dye uptake when compared to treated conditions with 140 mM KGlu alone (Figure 9A). Together, these results confirmed the effectiveness of two concentrations of KGlu to evoke dye uptake by Neuro2A cells expressing mPanx1. They verified that low and high ATP concentrations increase and reduce dye uptake, respectively, as previously shown in the oocyte expression system (Qiu and Dahl, 2009). Further, they confirm that probenecid (Prob) and brilliant blue food dye (BB FCF) are reliable blockers of mPanx1 in this cell model.

Using the identical experimental conditions, mPanx3-EGFP expressing cells without treatment showed a significant increase in dye uptake when compared to EGFP transfected Neuro2a cells (0.33 ± 3.20e^−3^, *n* = 157, *p*-value = 1.21e^−8^; Figure 9B), though it is important to note that this uptake is relatively lower than the average amount of uptake by mPanx1 under the same condition. Similarly, treatment of mPanx3 expressing cells with 50 mM KGlu (1.17 ± 0.013, *n* = 96, *p*-value = 1.12e^−18^), 140 mM KGlu (1.20 +/– 0.0037, *n* = 392, *p*-value = 5.12e^−40^) or 3 μM ATP (1.43 ± 0.012, *n* = 102, *p*-value = 4.69e^−23^) significantly increased dye uptake compared to untreated control cells expressing mPanx3-EGFP. Similar to mPanx1, application of 3 mM ATP inhibited the amount of dye uptake during stimulation with 140 mM KGlu when compared to stimulated controls (0.89 ± 0.010, *n* = 151, *p*-value = 1.20e^−10^; Figure 9B). Further investigation into blocking the mPanx3 channel showed that application of 500 μM Prob, prior to application of KGlu, efficiently inhibited dye uptake (0.46 ± 0.0077, *n* = 407, *p*-value = 2.16e^−12^), however, application of 10 μM BB FCF significantly increased mPanx3 channel activity (2.33 ± 0.022, *n* = 407, *p*-value = 1.38e^−14^). These results further established mPanx3 channel properties *in vitro*, in response to stimulants and inhibitors that act upon mPanx1. In summary, this quantitative assessment allowed us to conclude that Panx1 and Panx3 share two significant properties, dye uptake, and ATP release, suggesting that Panx3 can at least in part functionally compensate for Panx1.

## Discussion

This research shows the expression and localization of three Panxs in the VNO of both wild-type and a well-established mouse model with genetic ablation of mPanx1. Both mPanx1 and mPanx3 show differential expression in juvenile and adult mice, with a remarkable upregulation of Panx3 in the VNO—specifically in areas with no Panx1 expression after genetic ablation. Panx2 was ubiquitously distributed in the VNO, but not differentially regulated. The compensatory regulation of Panx3 in Panx1^−/−^ mice raised the question whether Panx3 forms channels, which, at least in part, can compensate for the loss of Panx1. Using another established model, Neuro2A cells expressing mPanx1 or mPanx3, channel function was demonstrated using two independent methods exhibiting that Panx3 could take up dye, release ATP, and respond to Panx1 blockers—providing the first evidence that Panx3 is forming functional channels with properties similar to Panx1.

### Pannexins in the Mouse VNO

Extending our previous research on Panxs in olfaction— suggesting indirect or even negligible roles of Panx1 in primary olfaction (Kurtenbach et al., 2014), we have shown in this study the expression of mRNA and protein localization of the three Panxs in the VNO. The results are different to our findings in the OE of mice, which express mPanx1, low levels of mPanx2, but no mPanx3 mRNA. Also, the IHC of the OE revealed a significant, but restricted, labeling of the OSN axon bundles that project to the olfactory bulb, but lacked immunoreactivity in the OSNs themselves (Kurtenbach et al., 2014). Further, no compensatory regulation of Panx3 was found in the OE of Panx1^−/−^ mice. To our knowledge, only one other sensory organ, the cochlea, expresses all Panxs in the same tissue (Wang et al., 2009; Zhao, 2016), whereas other parts of the nervous system typically express Panx1 and Panx2, but lack Panx3 (Bruzzone et al., 2003). In fact, our previous studies of other sensory organs, including the eye (Ray et al., 2005; Dvoriantchikova et al., 2006), primary olfactory system (Kurtenbach et al., 2014), or lacrimal gland (Basova et al., 2017) showed either lack, or very low levels of Panx3 gene expression.

The localization of mPanx1, as well as mPanx3 in Panx1^−/−^ mice, is consistent with the requirement for ATP release in the VNO (see “Discussion” section below). The expression and localization of Panx3 in the VNO after loss of Panx1 is remarkable, since Panx3 expression has typically been limited to skin, bone and cartilage (Celetti et al., 2010; Moon et al., 2015; Oh et al., 2015; Caskenette et al., 2016; Ishikawa et al., 2016), skeletal myoblasts (Langlois et al., 2014) or low diameter arteries (Lohman et al., 2012). Multiple studies have highlighted the importance of VNO function in reproductive behaviors in mice. Hence, it makes sense that a compensation system has evolved to maintain its function in extreme circumstances, e.g., loss of an essential protein. Our behavior tests did not reveal major changes of Panx1 KO for behaviors associated with VNO function, showing that Panx3 might be able to compensate for Panx1 loss efficiently. We did find a significant increase in smell time of resident Panx1^−/−^ mice towards intruders. This difference is potentially an interesting observation because investigating and sniffing at intruders may also be influenced by memory deficits (Prochnow et al., 2012). Generally speaking, the availability of Panx3 KO mice (Moon et al., 2015; Abitbol et al., 2016) has allowed for the investigation of the role(s) Panx3 plays in the bone, with results suggestive of Panx3 regulating chondrocyte and osteoprogenitor cell proliferation and differentiation, long bone growth, and skeletal formation and development (Moon et al., 2015; Oh et al., 2015; Abitbol et al., 2016; Caskenette et al., 2016). However, the investigation of the roles of Panx3 functions beyond these tissues have yet to be reported, with this study being the first to implicate a role for Panx3 in a sensory system.

### What Is the Evidence That ATP Release Plays a Critical Role in VNO Function?

We propose that ATP release is the key physiological role served by Panx1 and Panx3 in the VNO. Gerhard Dahl and his coworkers were the first to demonstrate the role of Panx1 as a major ATP release site (Locovei et al., 2006, 2007). Since then, Panx1 mediated ATP release has been found in many tissues and cell types, substantiating that this mode of regulated ATP release, alongside other non-vesicular ATP release pathways, is important to achieve tissue function(s) (for review see (Dahl and Keane, 2012; Scemes, 2012; Dahl et al., 2013; Penuela et al., 2013; Good et al., 2015). It is important to note that ATP release via Panx1 has not consistently been found (Taruno et al., 2013), which may indicate that some tissues do not rely on Panx1 mediated ATP release to achieve function, or compensatory upregulation was not investigated.

Pheromones are detected by VSNs once they are forced into the lumen of the VNO by a vascular pump triggered by sympathetic stimulation of the Vidian nerve (Eccles and Eccles, 1981), where we see an expression of Panx1 and Panx3 in juveniles. This mobilization of chemical signals is due to the sympathetic nervous system initiating ATP release (Rummery et al., 2007), which is also extremely important in evoking neural contractions of the vascularized erectile tissue on the lateral side of the lumen for further chemical processing. The signal transduction cascade is complex in the VNO, as the neuroepithelium consists of multiple distinct populations of VSNs. However, recent advances have implicated that there are subzone-specific ligands and sensory transduction components that enable VNO subdivisions to control specific olfactory-mediated behaviors (Kumar et al., 1999; Oboti and Peretto, 2014; Pérez-Gómez et al., 2014). For example, research conducted by Leinders-Zufall et al. (2014) has already demonstrated that aggressive behavior toward intruder males requires sensory transduction from basal VSNs. This is a region in adults where we saw the expression of Panx1 and increased Panx3 expression in KOs. We propose that important paracrine signaling functions of ATP for signal transduction are mediated by Panxs. The observed lack of upregulation of alternative channels and receptors is evidence in support of this concept. Together, the significant expression of Panx1 and Panx3 in both the basal and apical regions of the adult sensory layer, implicates the involvement of Panxs in olfaction in the context of adult social behavior, whereas the more restricted expression found in juvenile mice is more likely to serve the biomechanical functions of the developing VNO. Once Panx1 and Panx3 single and double KO mice become more broadly available, it will become possible to address this knowledge gap in longitudinal studies from juvenile to adult stages.

### Is Panx3 a Channel That Can Compensate for Panx1?

This study provides evidence that Panx3 is an ATP release channel similar to Panx1, supporting two claims of ATP release via Panx3, suggestive of channel function, in cultured ATDC5 skeletal cells (Iwamoto et al., 2010) and human odontoblast-like cells (Fu et al., 2015). Further, Panx3, like Panx1, takes up dye in a linear relationship with protein expression, albeit at different efficiency, and in response to known Panx1 blockers. Several results are notable. Both ATP release and dye uptake through mPanx1 and mPanx3 were activated by stimulation with a medium (50 mM) and a high (140 mM) concentration of KGlu. The later concentration of 140 mM has been used repeatedly since the introduction to the field (Bao et al., 2004; Silverman et al., 2009), providing robust activation in our hands.

It is also noteworthy that blockers previously reported acting individually on Panx1 block Panx3 as well. This effect is most likely due to the structural homology of both proteins, which share a 73.25% similarity at the amino acid level, and that Panx1 and Panx3 channels can interact to some, albeit low, extent with no observed change to the channel function when intermixed (Penuela et al., 2009). Interestingly, BB FCF, a selective blocker of Panx1 channels (Wang et al., 2013), reliably blocked ATP release in both mPanx1-EYFP and mPanx3-EGFP expressing Neuro2a cells. However, it selectively affected only mPanx1 mediated dye uptake. At present, the reason for the difference in BB FCF blocking efficiency of mPanx3 during dye uptake vs. measuring ATP release is unclear. Although, we surmise that this model imitates the model *in vivo* by efficiently blocking mPanx1 channels *in vitro* and observing the enhanced activity of mPanx3. Despite the fact that the blocker pharmacology of Panx1 channels remains bizarre, as eloquently highlighted by Dahl et al. (2013), the distinct responses to BB FCF could be a starting point for isolating Panx1 and Panx3 channel activities separately. Ultimately, the observation that BB FCF can increase dye uptake provides a unique opportunity to determine the mode of interaction between BB FCF and protein targets by domain swapping experiments using chimeras of mPanx1 and mPanx3.

While we can provide evidence for Panx3 channel activities using two different methods, key biophysical properties accessible only through electrophysiological methods are lacking. However, our results point to the possibility that the biophysical properties of Panx3 channels can be investigated using similar procedures used for Panx1. It is very likely that these properties would be difficult to isolate when the cell culture model used expresses endogenous Panx1 or Panx3, in particular when the robust activity of Panx1 may mask those contributed by Panx3. Therefore, generating genetically engineered cell lines, with ablation of Panx1 and Panx3, for example by using Cas9/CRISPR or Transcription activator-like effector nucleases (TALEN), could help to overcome this knowledge gap. Together, our results point to the possibility that Panx3 channels are more subtle and challenging to study using traditional biomolecular techniques including electrophysiology, which is in line with the lack of data in the literature. On a similar note, more specific Panx blockers need to be found, which will allow distinguishing Panx1 from Panx3 functions *in vitro* and *in vivo*.

### Compensatory Regulations of Pannexins: Why and How?

The upregulation of Panx3 observed in this study, has also been found in the vasculature (Lohman et al., 2012) and skin (Penuela et al., 2014) of different knock out mouse models. This suggests that the mobilization of Panx3 expression due to the absence of Panx1, as well as the properties shared between Panx1 and Panx3, is not restricted to a single loss of function phenotype.

Why is Panx3 upregulation required in the absence of Panx1? We hypothesize that the VNO requires tight control of ATP concentrations in a physiological range as a means of supporting prominent roles in triggering innate behavioral responses, like aggressive and reproductive behaviors (Pérez-Gómez et al., 2014). Since these behaviors are critical for the survival as a species, Panx3 upregulation could represent a safeguarding mechanism of unknown complexity that is innate to the cell type affected by the genetic KO and able to compensate for the loss of Panx1 as a major ATP release site—important in paracrine signaling.

The molecular mechanism of the compensatory regulation is open to speculations. Comparative studies of wild-type and KO mice at the level of the transcriptome, epigenome, proteome, or by promoter studies are likely to provide insight into the underlying molecular mechanism. In addition, the availability of Panx1 (Bargiotas et al., 2011; Qu et al., 2011; Dvoriantchikova et al., 2012; Hanstein et al., 2013) and Panx3 KO mice (Moon et al., 2015; Abitbol et al., 2016), as either single or double KOs, will be critical in this process. Further, future studies are also likely to aid in clarifying other evidence for compensatory regulation of Panxs, like in the vasculature (Lohman and Isakson, 2014) and skin (Penuela et al., 2014). However, whether these examples of compensatory regulation share a common mechanism, or a cell type or tissue-specific mechanism, needs to be determined.

## Author Contributions

PW-F, SK, CZ performed experiments. VIS, PLC provided animals and materials. All authors took part in analyzing the data, writing and editing the manuscript. GZ developed the concept and coordinated the research. All authors approved the final manuscript.

## Conflict of Interest Statement

The authors declare that the research was conducted in the absence of any commercial or financial relationships that could be construed as a potential conflict of interest.

## Supplementary Material

FIGURE S1 Quantitative PCR analysis of total RNA isolated from Neuro2a cells. The average cycle threshold (Ct) values demonstrated the expected high (18 s RNA; Ct < 20) and medium (ß-actin/ACTB; Ct 20–30) expression levels for the reference RNAs. Mouse Panx1 and mPanx3 mRNAs were expressed at medium (mPanx1; Ct 20–30) and low levels (mPanx3; Ct > 30). The very low expression of mPanx3 was positively identified through melt curve analysis of the PCR generated amplicon (data not shown). No expression of Panx2 mRNA was detected. Values represent mean ± SD. Abbreviation: n.d. = not detected.

FIGURE S2 Expression of pannexin mRNAs in transiently transfected Neuro2a cells. QRT-PCR showed overexpression of mPanx1 and mPanx3 in Neuro2a cells transiently transfected with mPanx1-EYFP or mPanx3-EGFP. All experiments were performed in triplicates. Values represent mean ± SD.

FIGURE S3 Linear correlation analysis of fluorescent reporter expression and uptake of ethidium bromide. Normalizing the amount of dye uptake, the fluorescence of EtBr, to the amount of fluorescent reporter expression (mPanx1-EYFP and mPanx3-EGFP), reveals a strong and consistent linear relationship under control and stimulated conditions. **(A)** mPanx1 under DMEM (closed dots), *R*^2^ = 0.88, and mPanx1 with 140 mM KGlu stimulation (closed triangles), *R*^2^ = 0.89. **(B)** mPanx3 with DMEM (open dots), *R*^2^ = 0.90, and mPanx3 with 140 mM KGlu stimulation (open triangles), *R*^2^ = 0.91.

FIGURE S4 Summary of the dye uptake assay. This image represents how the dye uptake assays were conducted. In accordance with the outlined figure, media was changed to media lacking phenol red, and the plate was incubated for 15 min, before transfer to the microscope into a live cell imaging chamber. Next, media with 10 μM EtBr and a stimulant of choice were applied. For stimulation conditions, this was the start of tracking the amount of dye uptake. For blocking conditions, 5 min after applying 140 mM KGlu, blockers were applied along with EtBr and then dye uptake was tracked. To track dye uptake, images were taken at 1-min intervals for 20 min, and the accumulated fluorescence of EtBr was calculated over time.

FIGURE S5 Automated cell selection overlay. The image shown represents an example for mPanx1-EGFP transfected Neuro2A cells (green) captured during a dye uptake assay. Superimposed in red and numbered are the regions of interest captured by the automated cell selection routine developed using ImageJ plugins, which were used for calculation of integrated density values.

FIGURE S6 Depiction of methodology for ATP detecting luciferase assays. This image is a representation of how the ATP detecting luciferase assays were conducted. Following the figure, cultures were incubated with a blocker for 5 min before application of 140 mM KGlu for 10 min. Otherwise, cultures incubated for 10 min with either low or high concentration of KGlu. Then, the supernatant was collected, and pipette into a 96 well plate along with an ATP standard curve, where reaction solution was applied according to the manufacturer’s protocol and plates were placed into a microplate reader to determine luminescence—indicative of the presence of ATP.

FIGURE S7 Secondary antibody control for Immunohistochemistry (IHC). A section of the juvenile VNO was processed for IHC and incubated with secondary goat anti-rabbit Alexa Fluor 488 antibody (Invitrogen, Canada) only. The confocal image showing background signal was captured using the identical settings used in Figure 3. Scale bar = 200 μM.
