# Supplementary figures and images for: A Potential Compensatory Role of Panx3 in the VNO of a Panx1 Knock Out Mouse Model
Source: Front Mol Neurosci. 2018 Apr 26;11:135. doi: 10.3389/fnmol.2018.00135 (PMC5946002; doi:10.3389/fnmol.2018.00135)

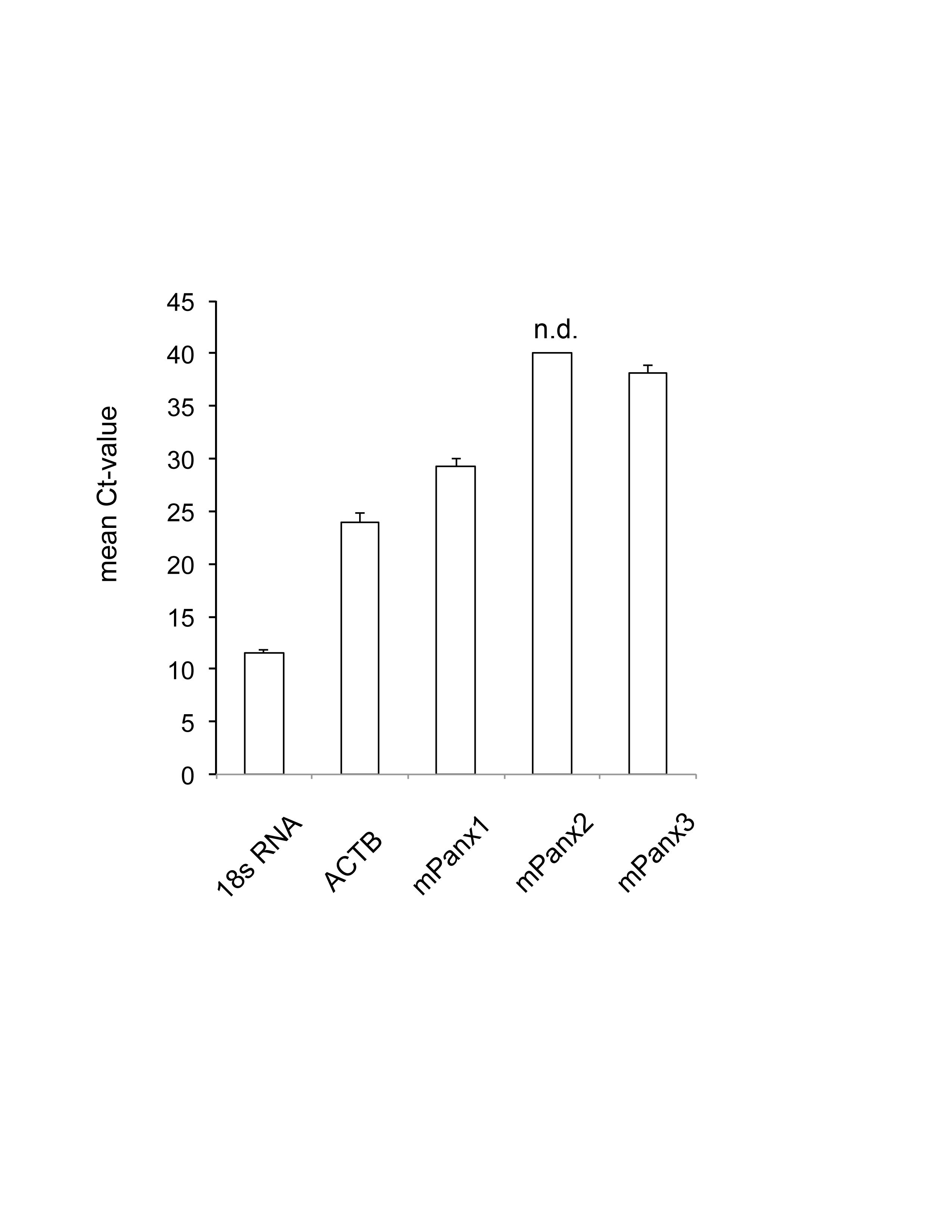

Supplement: FIGURE S1 — Quantitative PCR analysis of total RNA isolated from Neuro2a cells. The average cycle threshold (Ct) values demonstrated the expected high (18 s RNA; Ct < 20) and medium (ß-actin/ACTB; Ct 20–30) expression levels for the reference RNAs. Mouse Panx1 and mPanx3 mRNAs were expressed at medium (mPanx1; Ct 20–30) and low levels (mPanx3; Ct > 30). The very low expression of mPanx3 was positively identified through melt curve analysis of the PCR generated amplicon (data not shown). No expression of Panx2 mRNA was detected. Values represent mean ± SD. Abbreviation: n.d. = not detected. [file Image_1.JPEG]

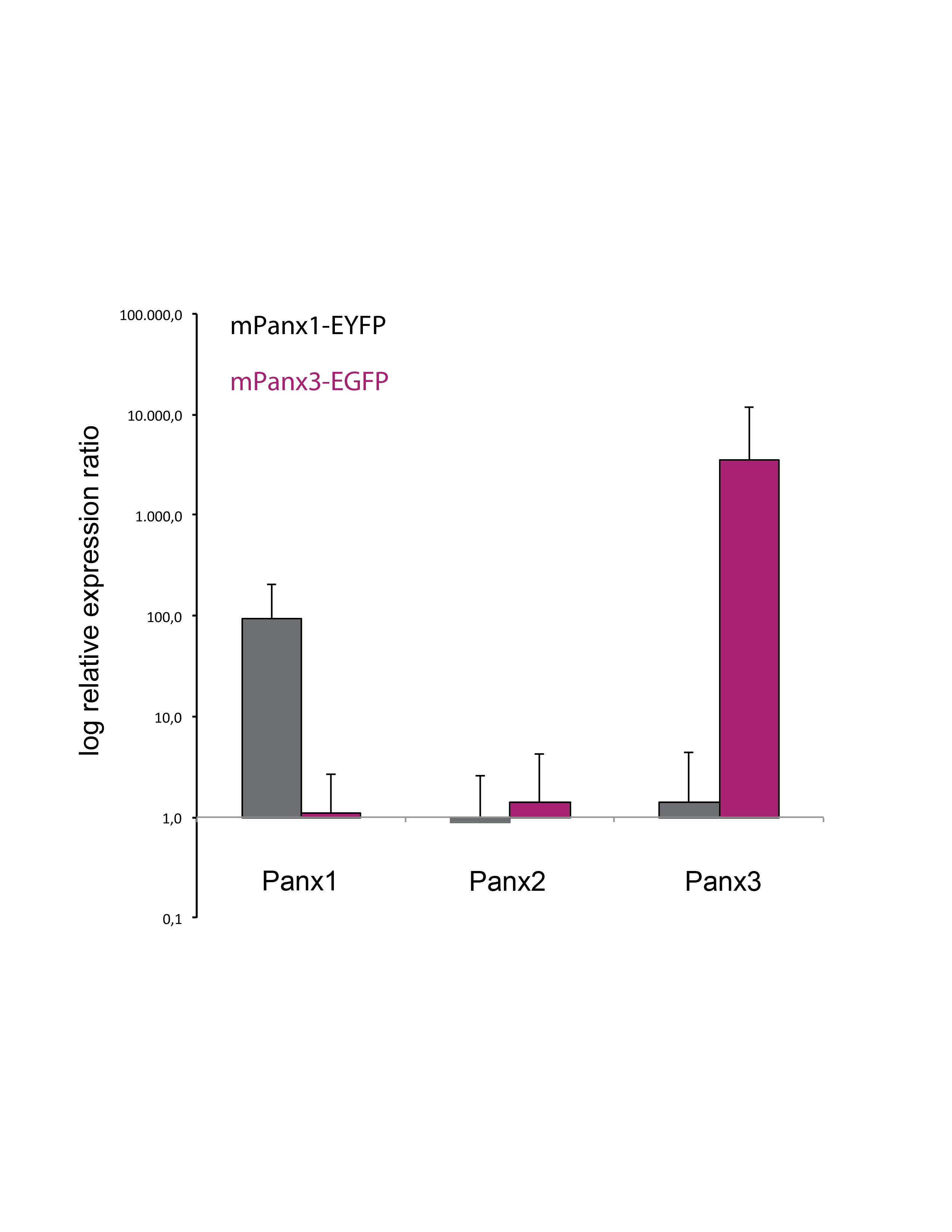

Supplement: FIGURE S2 — Expression of pannexin mRNAs in transiently transfected Neuro2a cells. QRT-PCR showed overexpression of mPanx1 and mPanx3 in Neuro2a cells transiently transfected with mPanx1-EYFP or mPanx3-EGFP. All experiments were performed in triplicates. Values represent mean ± SD. [file Image_2.JPEG]

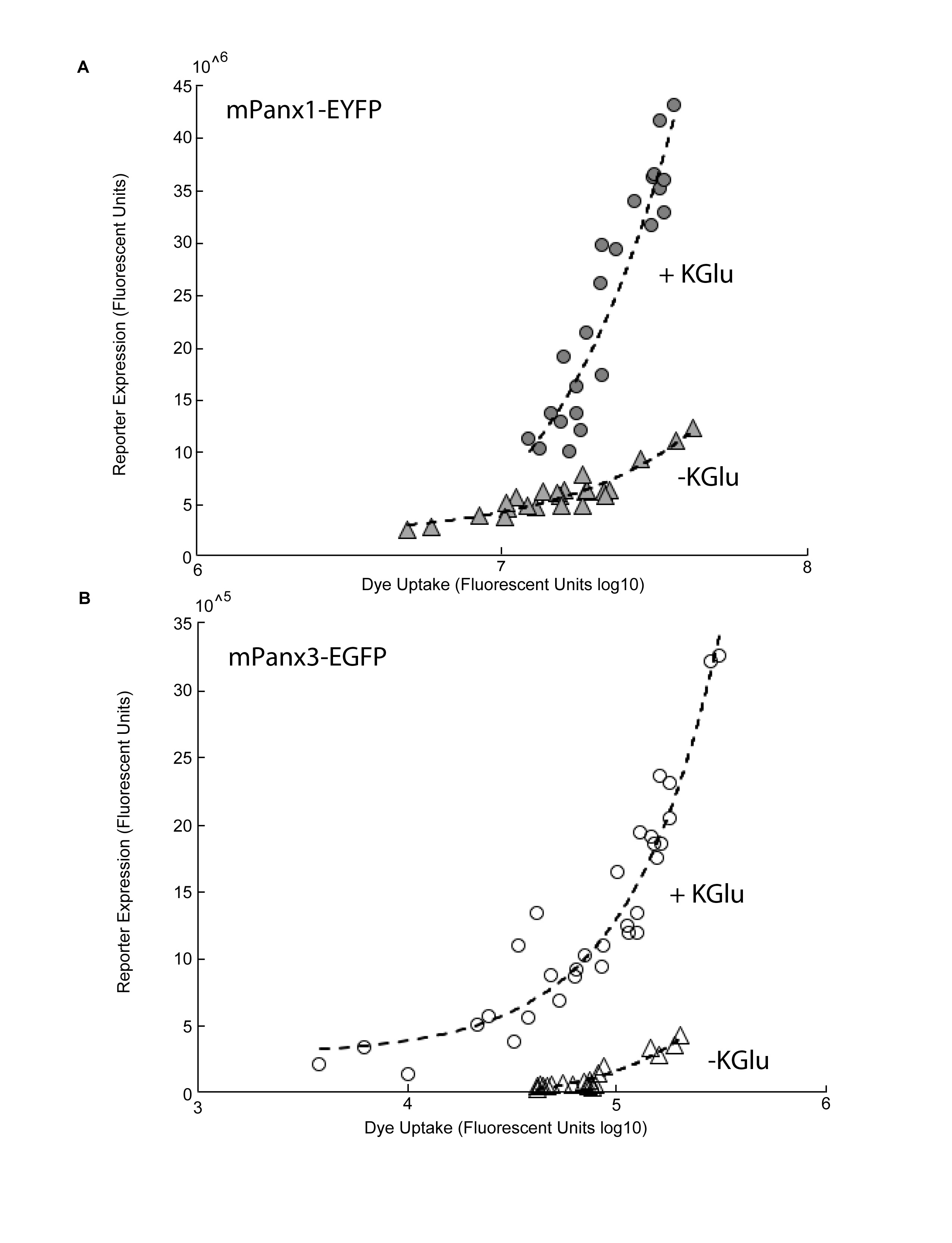

Supplement: FIGURE S3 — Linear correlation analysis of fluorescent reporter expression and uptake of ethidium bromide. Normalizing the amount of dye uptake, the fluorescence of EtBr, to the amount of fluorescent reporter expression (mPanx1-EYFP and mPanx3-EGFP), reveals a strong and consistent linear relationship under control and stimulated conditions. (A) mPanx1 under DMEM (closed dots), R2 = 0.88, and mPanx1 with 140 mM KGlu stimulation (closed triangles), R2 = 0.89. (B) mPanx3 with DMEM (open dots), R2 = 0.90, and mPanx3 with 140 mM KGlu stimulation (open triangles), R2 = 0.91. [file Image_3.JPEG]

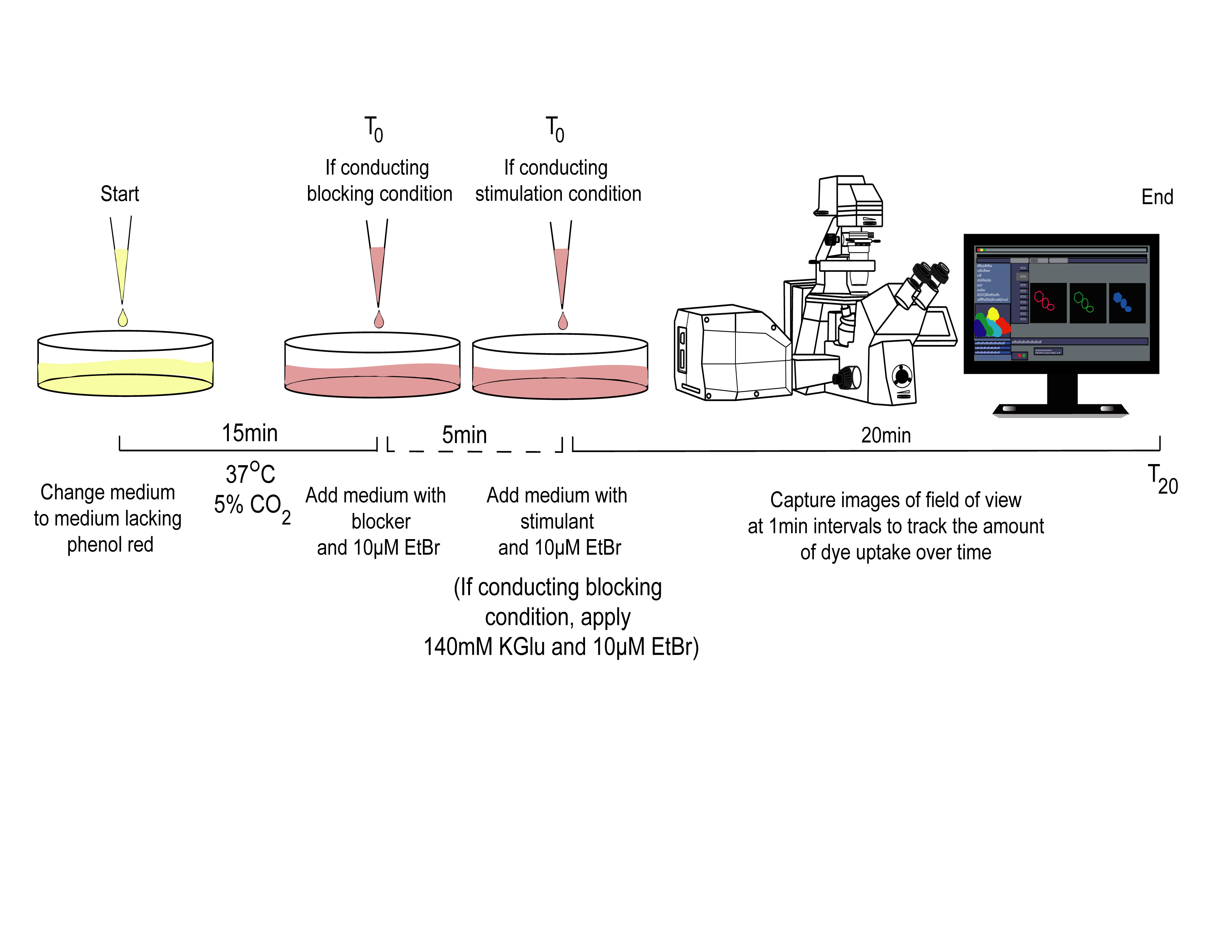

Supplement: FIGURE S4 — Summary of the dye uptake assay. This image represents how the dye uptake assays were conducted. In accordance with the outlined figure, media was changed to media lacking phenol red, and the plate was incubated for 15 min, before transfer to the microscope into a live cell imaging chamber. Next, media with 10 μM EtBr and a stimulant of choice were applied. For stimulation conditions, this was the start of tracking the amount of dye uptake. For blocking conditions, 5 min after applying 140 mM KGlu, blockers were applied along with EtBr and then dye uptake was tracked. To track dye uptake, images were taken at 1-min intervals for 20 min, and the accumulated fluorescence of EtBr was calculated over time. [file Image_4.JPEG]

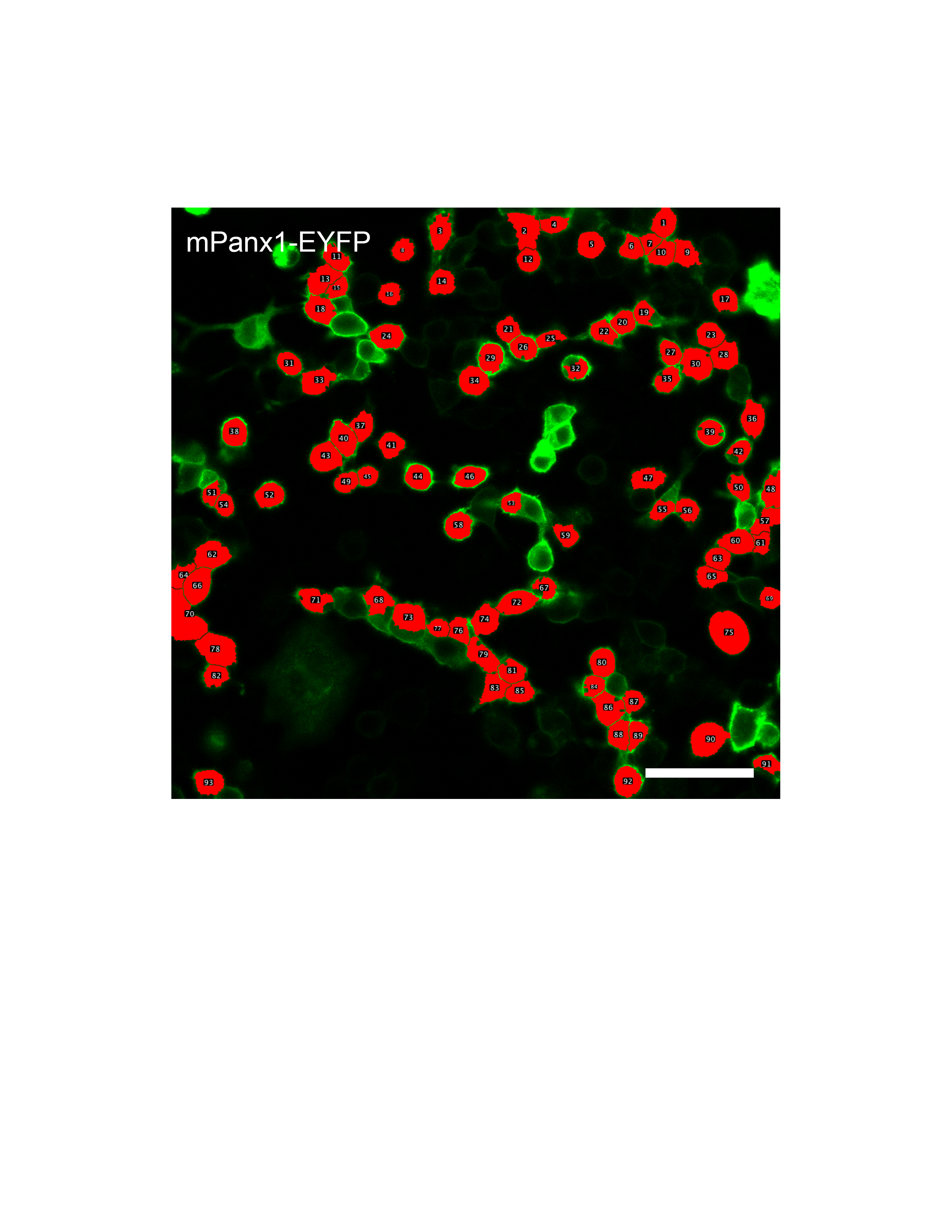

Supplement: FIGURE S5 — Automated cell selection overlay. The image shown represents an example for mPanx1-EGFP transfected Neuro2A cells (green) captured during a dye uptake assay. Superimposed in red and numbered are the regions of interest captured by the automated cell selection routine developed using ImageJ plugins, which were used for calculation of integrated density values. [file Image_5.jpg]

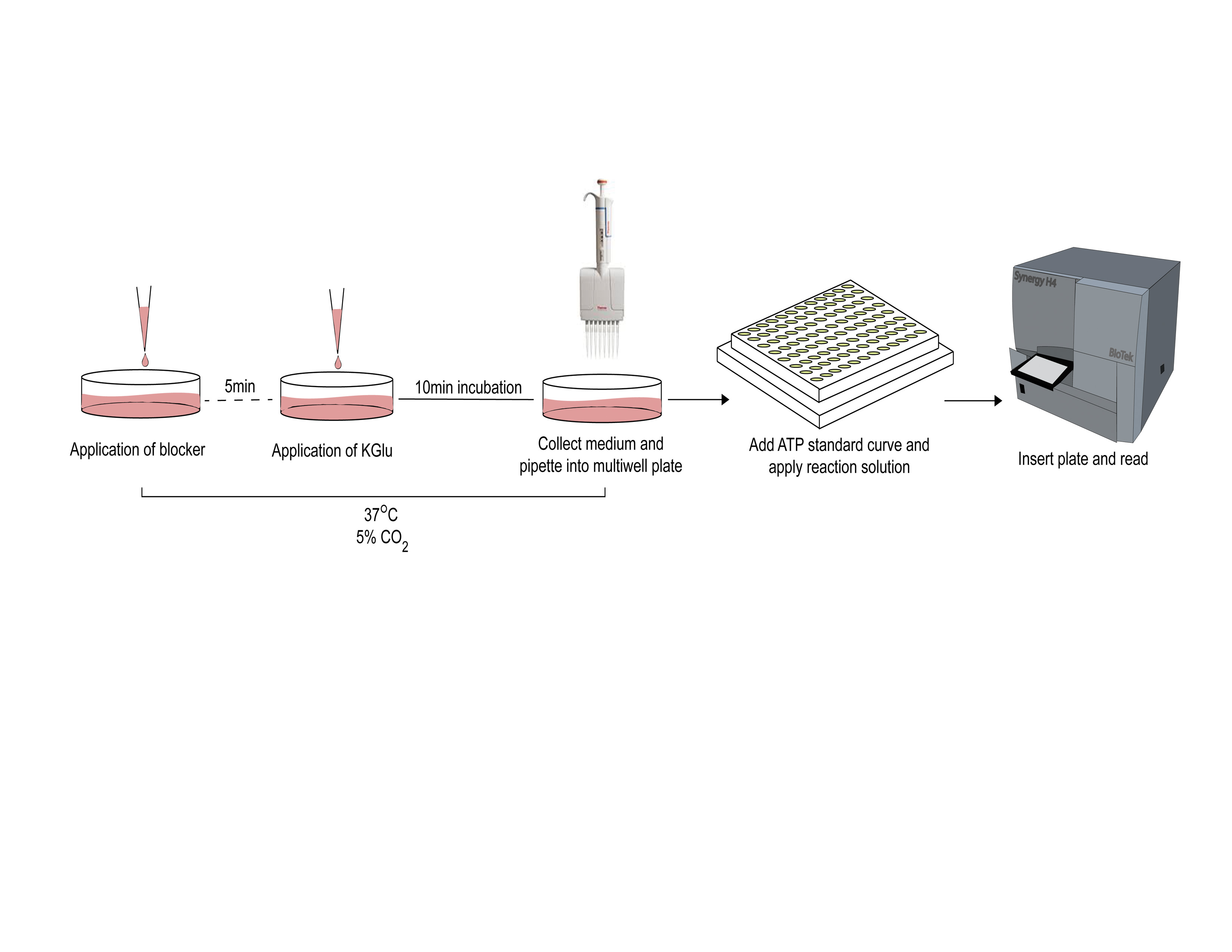

Supplement: FIGURE S6 — Depiction of methodology for ATP detecting luciferase assays. This image is a representation of how the ATP detecting luciferase assays were conducted. Following the figure, cultures were incubated with a blocker for 5 min before application of 140 mM KGlu for 10 min. Otherwise, cultures incubated for 10 min with either low or high concentration of KGlu. Then, the supernatant was collected, and pipette into a 96 well plate along with an ATP standard curve, where reaction solution was applied according to the manufacturer’s protocol and plates were placed into a microplate reader to determine luminescence—indicative of the presence of ATP. [file Image_6.jpg]

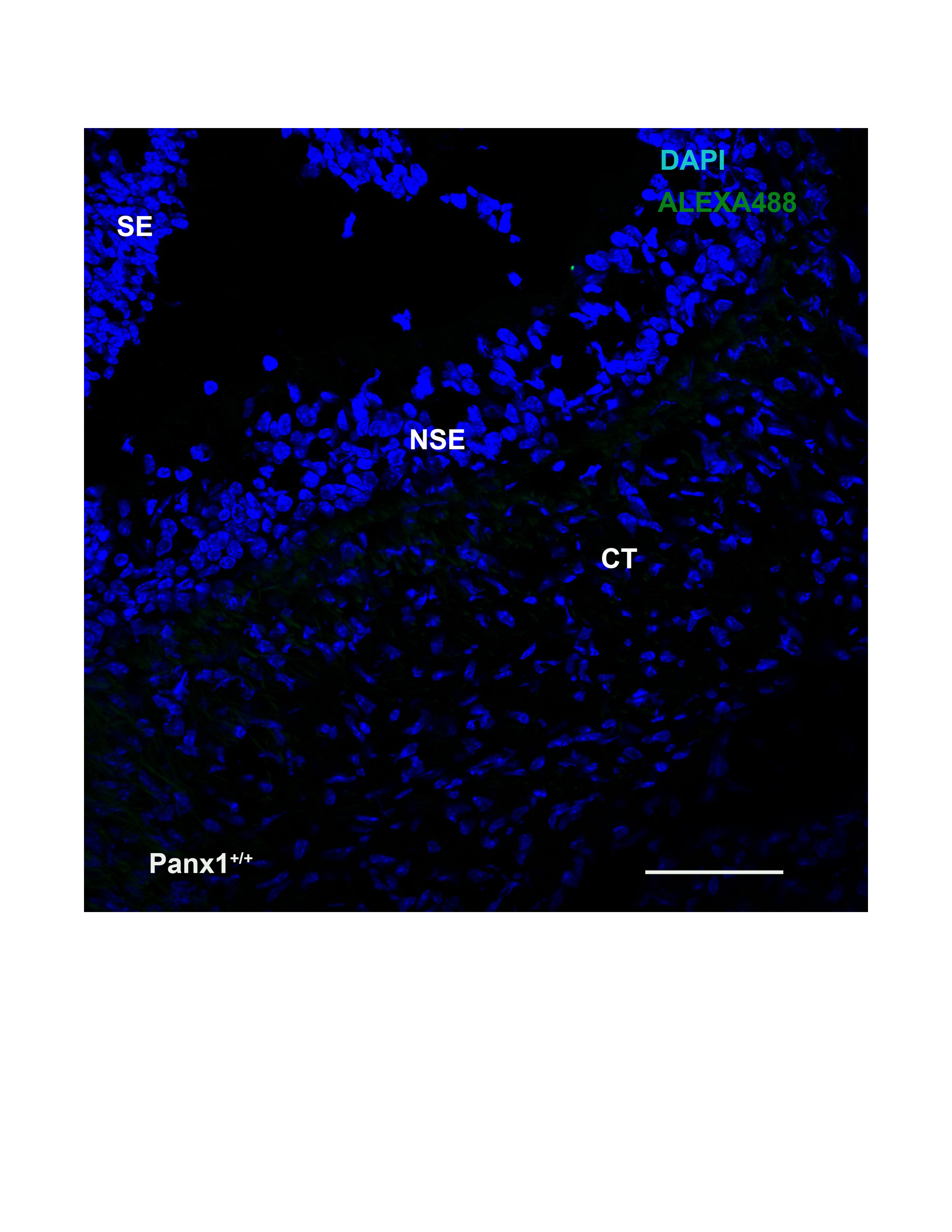

Supplement: FIGURE S7 — Secondary antibody control for Immunohistochemistry (IHC). A section of the juvenile VNO was processed for IHC and incubated with secondary goat anti-rabbit Alexa Fluor 488 antibody (Invitrogen, Canada) only. The confocal image showing background signal was captured using the identical settings used in Figure 3. Scale bar = 200 μM. [file Image_7.jpg]
